# Ex Vivo Drug Sensitivity of Pleural Effusion-Derived Cells from Lung Cancer and Pleural Mesothelioma Patients Is Linked to Clinical Response

**DOI:** 10.3390/cancers17142363

**Published:** 2025-07-16

**Authors:** Rita Hutyra-Gram Ötvös, Hanna Krynska, Greta Gudoityte, Marcus Skribek, Anca Oniscu, Olena Berkovska, Katharina Strauß, Jenny Zipprick, David Tamborero, Andrey Alexeyenko, Annica Karin Britt Gad, Brinton Seashore-Ludlow, Katalin Dobra

**Affiliations:** 1 SciLifeLab, 171 21 Solna, Sweden; rita.hutyragram@ki.se (R.H.-G.Ö.); greta.gudoityte@ki.se (G.G.); david.tamborero@ki.se (D.T.); andrej.alekseenko@scilifelab.se (A.A.); brinton.seashore-ludlow@ki.se (B.S.-L.); 2 Department of Oncology-Pathology, Karolinska Institutet, 171 64 Solna, Sweden; marcus.skribek@ki.se (M.S.); anca.oniscu@ki.se (A.O.); jenny.zipprick@gmail.com (J.Z.); annica.gad.2@ki.se (A.K.B.G.); katalin.dobra@ki.se (K.D.); 3 Medical Unit Head-Neck, Lung, Skin Cancer, Theme Cancer, Karolinska University Hospital, 171 76 Stockholm, Sweden; 4 Evi-Networks Consulting, 141 33, Huddinge, Sweden; 5 Department of Cell and Molecular Biology, Karolinska Institutet, 171 76 Solna, Sweden; 6 Department of Clinical Pathology and Cytology, Karolinska University Hospital, 171 77 Solna, Sweden

**Keywords:** lung cancer, mesothelioma, metastasis, pleural effusion, ex vivo drug sensitivity testing, functional precision medicine, personalized medicine, patient survival, targeted sequencing, network enrichment analysis

## Abstract

Background: Tumors of the pleura, such as metastatic lung cancer and mesothelioma, are amongst the most lethal and therapy-resistant tumors. The first manifestation of the disease is often pleural effusion, the first available material for diagnosis. The five-year survival rate is exceptionally low, around 10–20%, and only a small proportion of patients harbor mutations that allow targeted treatments. Almost all patients develop resistance to treatment, which is often palliative. There is therefore an urgent need to refine the selection of drugs and patients for personalized treatment. Methods: We isolated and cultured cells from pleural effusions in 3D cell aggregates and compared their drug sensitivity ex vivo to the clinical response to the same chemotherapeutic agents, combined with targeted sequencing and network analysis. Results: The ex vivo drug response showed a positive correlation with the treatment response and survival of patients in the clinic, with a stronger link to overall survival than to progression-free survival. Cryopreserved cells showed a similar response to freshly collected cells from the clinic. Conclusions: The findings advance the field of ex vivo screening and present an opportunity to combine strategies for functional precision medicine with comprehensive characterization of disease for improved treatment and future management of lung cancer.

## Simple Summary

Tumors of the pleura, such as lung cancer metastasis and pleural mesothelioma, are amongst the most lethal and therapy-resistant tumors, for which there is an urgent need to develop personalized treatment. A comparison between the ex vivo response of pleura-derived cells and patient outcomes in the clinic, combined with targeted sequencing and network analysis suggests that ex vivo drug screening adds value by predicting the clinical outcome, especially if combined with a molecular analysis and a comprehensive characterization of the disease. The findings provide valuable knowledge for the implementation of ex vivo testing approaches in the future and inform personalized treatment strategies for tumors in the pleura.

## 1. Introduction

Lung cancer represents a wide and complex spectrum of heterogeneous tumors [1]. Non-small-cell lung cancer (NSCLC) accounts for approximately 85% of cases, while small-cell lung carcinoma (SCLC) represents the remaining 15% [2]. Pleural mesothelioma (PM), another aggressive malignancy, develops in the mesothelial lining of the pleural cavity that surrounds the lungs [3]. These cancers are often diagnosed at an advanced stage, which limits the effectiveness of standard treatments [2,3]. The five-year survival rate of PM and metastatic lung cancer is exceptionally low, and only a small proportion of patients harbor mutations that allow targeted therapy [3,4]. A common manifestation of advanced, metastatic NSCLC and primary PM is the accumulation of a malignant pleural effusion (PE), which is used for diagnosis and molecular testing to guide personalized treatment plans based on systemic-, targeted- or immunotherapy [5,6].

Over the last decade, ex vivo drug sensitivity testing (DST) has emerged as a promising tool to guide clinical decision-making. In recent years, research has highlighted the importance of using fresh tumor cells to preserve tumor heterogeneity and has shifted from 2D to 3D cell models to better mimic the tumor microenvironment [5,7,8,9,10]. Tumor cells in PE offer the advantage of being easily collected through thoracocentesis, a minimally invasive procedure routinely performed to relieve the patient’s symptoms [5]. More recent studies have developed models of higher complexity by including an extracellular matrix or developing organoid models to better mimic the tumor environment [5,7,8,11]. However, more complex models do not necessarily show a better prediction of clinical outcomes and are difficult to include in clinical workflow within a relevant timeframe [12]. Nonetheless, several studies have demonstrated a general correlation between the DST approach and clinical outcome, highlighting its potential to optimize therapies by reducing unnecessary toxicity and, perhaps more importantly, improving patient outcomes [6,8,9,10,11,12].

Precision medicine relies on comprehensive characterization of an individual patient’s disease, integrating genomic and transcriptomic profiles to identify the most effective treatment for each patient [13]. In addition, ex vivo DST can provide functional sensitivity data that can help identify effective treatments for individual patients, overcome drug resistance and improve patient survival. Here, we adapt a recently developed scalable functional precision medicine platform [14] to the real-world clinical setting of lung cancer, where we assess the response of patient-derived cells from pleural effusion to clinically relevant chemotherapeutic agents. This is a double-blind retrospective observational study with ex vivo testing and an average of 10 months’ clinical follow-up of 21 patients. Tumor cells obtained from pleural effusions are grown as 3D aggregates, followed by quantification of their sensitivity to clinically relevant drugs. Our workflow, completed within 10 days, maintains the biological relevance of 3D cultures while preserving simplicity and reproducibility.

Our findings highlight the potential of ex vivo testing for the future development of personalized medicine strategies for pleural tumors.

## 2. Materials and Methods

### 2.1. Patient Population, Study Design and Specimen Collection

This retrospective double-blind observational cohort study included patients with malignant effusion who were diagnosed at Karolinska University Hospital over two periods, from 1 April 2021 to 17 November 2021 and from 18 January 2023 to 13 January 2025. Patients were eligible if they had malignant pleural effusion, corresponding to primary pleural mesothelioma (PM) or metastatic NSCLC. The inclusion criteria were defined as fresh effusion left after the diagnostic procedure, corresponding to at least 50 mL and containing more than 20% tumor cells. In addition, the diagnostic inclusion criteria for NSCLC were TTF-1 or p40 positivity and for PM, calretinin and epithelial membrane antigen (EMA) positivity and negative nuclear staining for breast-cancer-associated protein-1 (BAP-1) or negative cytoplasmic staining for desmin. The exclusion criteria were inadequate sample quality for immunophenotyping or molecular characterization, patient deceased prior to the completion of the diagnostic work-up or the diagnosis was not conclusive. Clinical data were collected from electronic medical records by a clinical oncologist (M.S.).

This study was approved by the Swedish Ethical Review Authority (approval number: 2009/1138-31/3, amendment 2020-05102) and conducted in accordance with the Declaration of Helsinki. The treatments followed standard treatments and doses according to international guidelines at the treating physician’s discretion. The clinical stages were classified using the 8th edition of the TNM classification.

### 2.2. Assessment of Clinical Outcome

Progression-free survival (PFS) was defined as the time from the start of treatment to the date of radiological or clinical progression. Overall survival (OS) was defined as the time of cancer diagnosis to death from any cause, with living patients censored at the last follow-up. In order to specifically capture survival from malignant pleural effusion, we have calculated the OS and focused on therapies from this time point to minimize bias. Given that radiological assessments were not consistently available and RECIST version 1.1 is not routinely applied in clinical practice, clinical benefit, based on symptom improvement and clinical judgment, was used as a surrogate marker of treatment response.

### 2.3. Preparation of Specimens and Experimental Design

Routine stains were performed at the Laboratory of Clinical Cytology at Karolinska University Hospital. For each patient sample, May–Grunwald–Giemsa (MGG), p40 and breast-cancer-associated protein 1 (BAP1) stains and dual-marker immunostainings with BerEp4 and calretinin, desmin and epithelial membrane antigen, thyroid transcription factor-1 (TTF-1) and napsin A were prepared. The percentage of cancer cells per total cell count was estimated by light microscopy. All the samples with a percentage higher than 25% were considered sufficient for drug sensitivity testing.

Upon arrival at the experimental lab, the pleural effusions were centrifugated at 800× *g* for 10 min at 4 °C to isolate cellular fractions. The cell pellet was resuspended in 1× ACK lysis buffer (Invitrogen, Waltham, MA, USA) and incubated at room temperature for 5 min to remove red blood cells. RPMI medium (Biowest, Sigma-Aldrich, Burlington, MA, USA) supplemented with 10% FBS (Gibco, Thermo Fisher Scientific, Waltham, MA, USA) was added, followed by centrifugation at 500× *g* for 5 min. After, the cells were resuspended in RockiT media and filtered through a 70 µm strainer (pluriSelect, Leipzig, Germany) and counted [14]. The extracted cells were either used immediately for drug sensitivity testing or cryopreserved in 2× freezing media containing 0.37% methyl cellulose (Sigma-Aldrich, Burlington, MA, USA), 16% DMSO (Sigma-Aldrich) and 84% FBS (Sigma-Aldrich). A cell pellet of 1 million cells was washed in PBS to remove residual fluids and stored at −80 °C until further processing for whole genome analysis.

### 2.4. Cell Culture

For the 3D culture, patient-derived cancer (PDC) cells were cultured in RockiT media [14] in U-bottom ULA 384-well plates (Corning, New-York, NY, USA). Commercially available cell lines (CCLs) HCC827 (RRIF: CVCL_2063) and H1869 (RRIF: CVCL_1500) were grown in RPMI (Biowest) medium supplemented with 1% fetal bovine serum (Gibco) and 1× antibiotics Streptomycin/Penicillin, (Gibco). All the cells were maintained in a 5% CO_2_-humidified incubator at 37 °C. The cell lines were screened for mycoplasma contamination (Lonza, Basel, Switzerland), and the CCLs were validated by short-tandem repeat (STR) sequencing (Eurofins, Luxembourg City, Luxembourg), while the PDCs were validated by target sequencing as previously described [15]. The CCLs were a kind gift from Lukas Orre, Karolinska Institutet, Solna, Stockholm.

### 2.5. Ex Vivo Drug Treatment

**Figure 1:**
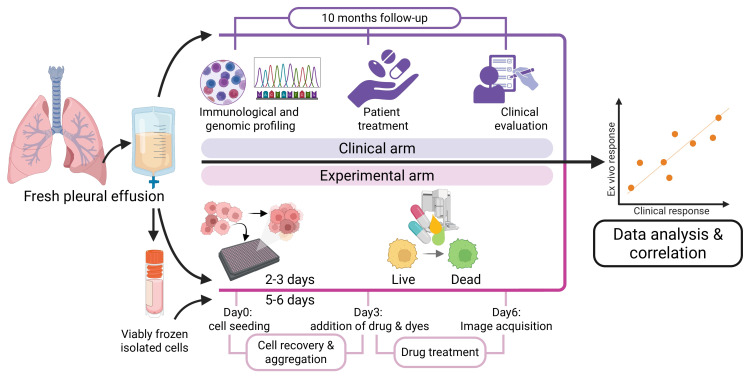
Experimental approach used to compare ex vivo to clinical outcome. Cryofrozen and thawed, or fresh patient-derived pleural cells cultured in 3D aggregates and analyzed for ex vivo sensitivity to drugs (pink area), in parallel with profiling and analysis of patient treatment and outcome (purple area), followed by correlation analysis of ex vivo to clinical response.

Cells isolated from pleural effusion were used for functional drug testing, following the modified DET3CT protocol based on Åkerlund et al. [14], adapted for viably frozen cells (Figure 1, experimental arm). Briefly, 3500 cells per well were seeded into U-bottom plates, as described above, centrifuged at 200× *g* for 1 min and incubated in a humidity box at 37 °C, 5% CO_2_. For viably frozen samples, the medium was replaced after 2–3 days to ensure cell recovery. Both the fresh and frozen samples were treated after 3 additional days in culture. The drugs were pre-spotted in 384-well plates (Greiner, Kremsmünster, Austria) and transferred to the cell plates using Apricot^®^S3 (SPT Labtech, Melbourne, UK). Spheroids were imaged at 3 and 72 h post-treatment using high-content screening system Opera Phenix (Revvity, Waltham, MA, USA). Imaging was performed with a 10× objective, capturing 1 field of view per well with 12–15 z-tack planes. Images were acquired using three channels: one brightfield and two fluorescent channels to detect live (TMRM, Thermo Fisher Scientific) and dead (SYTOX^TM^ Green, Thermo Fisher Scientific) cell signals.

### 2.6. Design of the Drug Library

A drug library of 14 FDA-approved drugs based on the national recommendation for stage IV NSCLC and PM was tested for single drug efficacy at 5 concentrations over a clinically relevant range from 1 nM to 10,000 nM (Supplementary Table S1). Double drug combinations (n = 3) were selected according to clinical importance in NSCLC and MM and were tested on 21 patient PE samples (Supplementary Table S2).

### 2.7. Drug Screening Data Analysis

Th acquired images were analyzed using Harmony software v5.2 (Revvity) following the image analysis pipeline as described by Åkerlund et al. [14]. The assay quality was evaluated based on the ability to discriminate between positive and negative controls, defined as cells treated with BzCl and DMSO, respectively. Drug response scores (DSS) were calculated using Breeze software (v2.0) (FIMM) [16] based on live spheroid volume data and DSS calculation. Drug synergy, as ZIP synergy scores, was assessed using the R package “synergy finder” (v3.16.0).

### 2.8. DNA Extraction and Targeted Sequencing Panel

Genomic DNA was extracted from the cell pellets obtained from patients LuCA008, LuCA025, LuCA057, LuCA058 and LuCA059 using the AllPrep DNA/RNA Mini Kit (QIAGEN, Hilden, Germany), following the manufacturer’s protocol to ensure high-quality DNA suitable for sequencing applications. The DNA concentration was quantified using Qubit 4 (Thermo Fisher Scientific, Waltham, MA, USA). Targeted sequencing was performed by the Clinical Genomics Stockholm facility at SciLifeLab using panel GMCKsolid (v4.2) and standardized pipelines for high-throughput library preparation (KAPA Library preparation using ODTC and MPH96, v. 10.4.29), sequencing (NovaSeq X v. 10.4.42) and variant calling with Balsamic software (v16.0.1) for genome version hg 19.

### 2.9. Network Enrichment Analysis and Prediction of Cancer Driver Genes

In order to evaluate and rank the mutated genes according to potential driver roles, we used the NEAdriver method, which was previously tested, calibrated and validated on ten large cancer cohorts [17,18]. NEAdriver implements the approach of network enrichment analysis, NEA, to evaluate the significance of network interactions by accounting for node degrees of interacting genes. NEAdriver uses the following two separate channels: (1) MutSet, evaluating driver roles for each mutation locally, i.e., by connectivity to other genes mutated in the same tumor genome (Supplementary Figure S1A); and (2) PathReg, predicting “driverness” globally, i.e., from the connectivity of the gene to a number of informative pathways included in the pre-trained machine learning model (Supplementary Figure S1B). Both MutSet and PathReg produce scores which are then converted to q-values. The final step integrates the latter into a combined q-value, which conveys the probability that driverness was assigned to a given mutation wrongly, i.e., neither MutSet nor PathReg provided real evidence for its functional involvement in the patient’s cancer genome. The advantage of the NEAdriver method is that it is not dependent on mutation frequency across a cancer cohort and can thus be applied to individual samples. As a result, both rare and common mutations can be detected with equal efficiency without collecting a large cohort. A possible drawback is that NEAdriver is less sensitive regarding driver genes, such as TP53 or EGFR, which can confer cancer phenotype without interacting with other mutations specific to individual genomes. The five LuCa genomic samples subjected to the panel-targeted sequencing were represented by sets of mutations marked by BALSAMIC as variant calling pipeline, as likely affecting respective peptides. For “protein-coding” genes, we required non-empty fields “exon”, “Sift” and “PolyPhene”. This resulted in multiple variants of mutated genes per sample. A global interaction network for NEAdriver analysis was created by merging all the links reported as experimentally validated PathwayCommons resource (v. 12) [19] with the top 1,000,000 links predicted by STRING resource (v. 12) [20], which resulted in 1,794,246 links between 21,073 gene symbols. For training the PathReg model, we used 658 “KEGG medicus” pathways, which are, on average, more compact than the pathways from the (no longer supported) traditional KEGG collection [21]. As input variables to be predicted by the trained PathReg model, we submitted the MutSet scores calculated for all the mutated genes in the LUAD TCGA cohort [22]. The resulting model was thus optimized for predicting PathReg scores for novel mutations in cancer genomes similar to LUAD.

### 2.10. Correlation Analyses

Correlation analyses of the responses were performed on patients who received corresponding treatment in the clinical setting and for whom the screening platform provided evaluable drug response data. For patients who had received triple therapy, we used the DSS value for carboplatin/pemetrexed at 10 µM of carboplatin. The correlation between the ex vivo and clinical data was considered strong if the correlation coefficient was between ±0.5 and ±1. Values between ±0.30 and ±0.49 were considered moderate, while below +0.29 was considered a weak correlation. A value of zero signaled no correlation.

### 2.11. Statistical Analysis

Descriptive statistics were used to summarize the patient characteristics. Median PFS and OS were calculated to describe survival outcomes. Two-sided Spearman correlation was applied to evaluate the correlation between DSS and PFS or overall survival from pleural effusion recovery (OS PE). A Kaplan–Meier curve was generated after splitting the cohort into two sub-groups based on the median DSS. For relevant matching correlates of drug sensitivity/resistance, corresponding significant *p*-values from the GDSC1, GDSC2 and CTRPv2.0 datasets were identified after Bonferroni correction for multiple testing, then combined with the *p*-value from our ex vivo screen using Fisher’s formula for combining multiple independent tests. The statistical analyses were conducted using GraphPad Prism 10.2.2 version.

## 3. Results

### 3.1. Patient Characteristics

**Figure 2:**
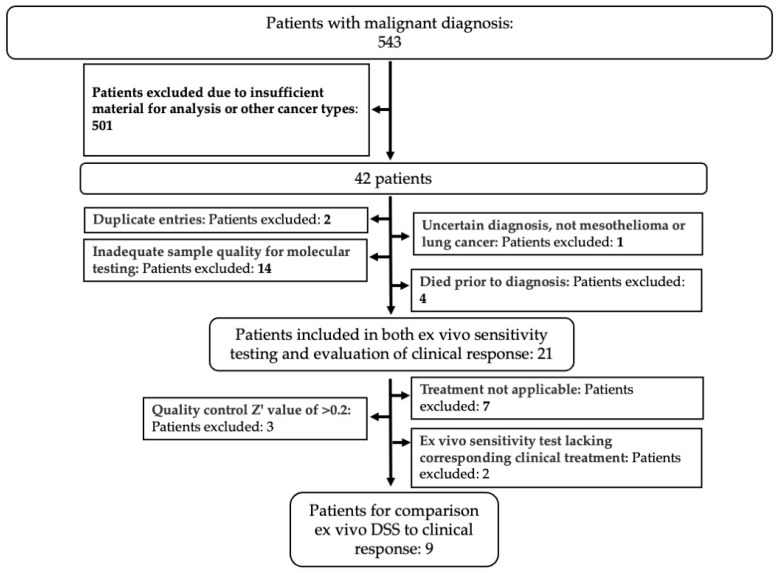
Selection of patient cohort. Patients with a malignant effusion diagnosed at the Karolinska University Hospital were selected based on inclusion and exclusion criteria (bold text) as indicated, to obtain the cohort of patients used in the study.

Out of 543 patients diagnosed with malignant effusion at Karolinska University Hospital, 21 patients were included in the final analysis based on the inclusion criteria shown in Figure 2. The median follow-up time was 10 months, with 12 female (57%) and 9 male (43%) patients. Most patients were former smokers (11 patients, 57%), while 7 (33%) had never smoked and 3 (14%) were current smokers. Adenocarcinoma was the predominant histology in 19 patients (90%), while 2 patients (10%) had mesothelioma. We observed that the PD-L1 expression was low or intermediate in 15 patients (72%) and high in 2 (9%), while 4 (19%) had not been tested. Actionable genetic mutations were present in 12 (57%) of the cases, and 11 (52%) patients presented with pleural effusion at cancer diagnosis (Table 1). At the time of data cut-off, 14 (67%) patients had died (Table 1).

### 3.2. Patient Outcomes

The patient cohort was thereafter analyzed with regard to diagnosis, received therapy and clinical response, as summarized in Table 2. We can further observe that 9 (42.9%) patients received targeted therapy, most commonly osimertinib (24%). Other targeted agents included alectinib, selpercatinib, pralsetinib and the combination of dabrafenib and trametinib (Table 3). Combination chemotherapy regimens were used in 5 (24%) patients, primarily platinum-based doublets with immune checkpoint inhibitors. One-third of the cohort (33%) did not receive any treatment (Table 2). At treatment initiation, 12 (57%) patients had Eastern cooperative oncology group performance status (ECOG PS) 0–1, while 3 (14%) had performance status (PS) 3. Among the 14 patients who received treatment, clinical benefit was observed in 10 (71%), while 3 (21%) showed no benefit and 1 patient was not evaluable. The median PFS for the cohort was 6 months, with similar outcomes in patients with and without targetable mutations. The median OS was 10 months in the overall cohort, 14 months for patients with actionable mutations and 8 months for those without. From the time of malignant pleural effusion diagnosis, the median OS was 3.0 months overall, 3.5 months in patients with actionable mutations and 3.0 months in those without (Table 2).

### 3.3. Adaptation of the Ex Vivo Drug Sensitivity Profiling Platform for Pleural Effusions

**Figure 3:**
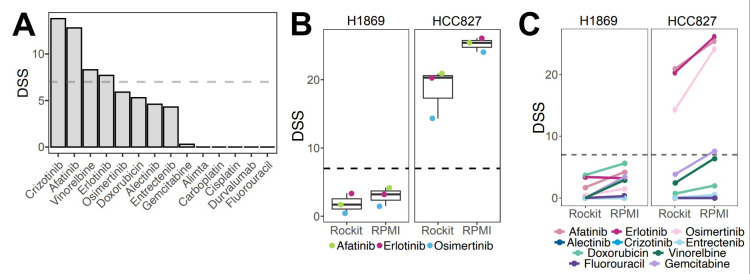
Adaptation of the drug screening platform to the PE samples using cell lines and LuCa11 cells obtained from pleural effusion. (**A**) Drug responses, as DSS scores, for LuCa011-PDC. (**B**) Effects of EGFR inhibitors on two lung cancer cell lines in RockiT or RPMI media; colored dots represent EGFR inhibitors. (**C**) Drug response of lung cancer cell lines cultured in Rockit and RPMI media, treated with single drugs.

Here, we adapted the DET3CT platform, originally tested on ovarian cancer tissue and ascites [14], to assess drug response in lung cancer and mesothelioma patients using PE. The assay was originally tailored to fresh (hereafter referred to as “original”) and viably frozen PE samples. To assess drug response, we tested 14 drugs used in lung cancer management based on the national recommendations for treatment of stage IV lung cancer (National Clinical Cancer Care Guidelines), including chemotherapeutics, targeted therapies and immunotherapy (Supplementary Table S1). In addition, we included clinically relevant drug combinations of carboplatin and alimta, gemcitabine or vinorelbine. We first used the adapted DET3CT assay on patient-derived cancer (PDC) cells LuCa011, which was generated from PE. LuCa011 harbors several genetic mutations associated with NSCLC (Supplementary Table S3), as well as high CNV burden (Supplementary Figure S2). LuCa011 was successfully screened with the DET3CT assay, resulting in a good assay quality (Z′ = 0.66) (Supplementary Table S4). Among all the drugs, ALK inhibitor crizotinib, microtubule inhibitor vinorelbine and EGFR inhibitors erlotinib and afatinib demonstrated moderate to high drug efficacy (DSS > 7) (Figure 3A). It is important to note that the sample culturing media used in these assays contains epidermal growth factor (EGF), which may influence cell drug response to EGFR inhibitors in particular [23]. To determine the potential media-related effects, we evaluated drug responses in two commercial lung cancer cell lines: HCC827, harboring an amplification of EGFR and exon 19 deletion [24], and H1869, a squamous cell carcinoma cell line with unknown EGFR status [25]. Both cell lines were tested in RockiT media and the recommended standard culture media. As expected, our results confirm that HCC827 cells were sensitive to all the tested EGFR inhibitors present in the LuCa library, while H1869 showed resistance to these drugs. In addition, the drug response trends remained consistent across the culturing conditions with no statistically significant difference observed in the HCC827 or H1869 cell lines (*p* = 0.07697 or *p* = 0.3891, respectively) (Figure 3B). Moreover, there was no significant impact in drug response against the other tested drugs (Figure 3C). Altogether, we successfully adapted the DET3CT platform for use with PE samples and demonstrated that the experimental conditions used do not significantly influence key drug responses.

### 3.4. Functional Drug Testing of Pleural Effusions Demonstrated Feasibility and Clinically Relevant Patient-Specific Responses

**Figure 4:**
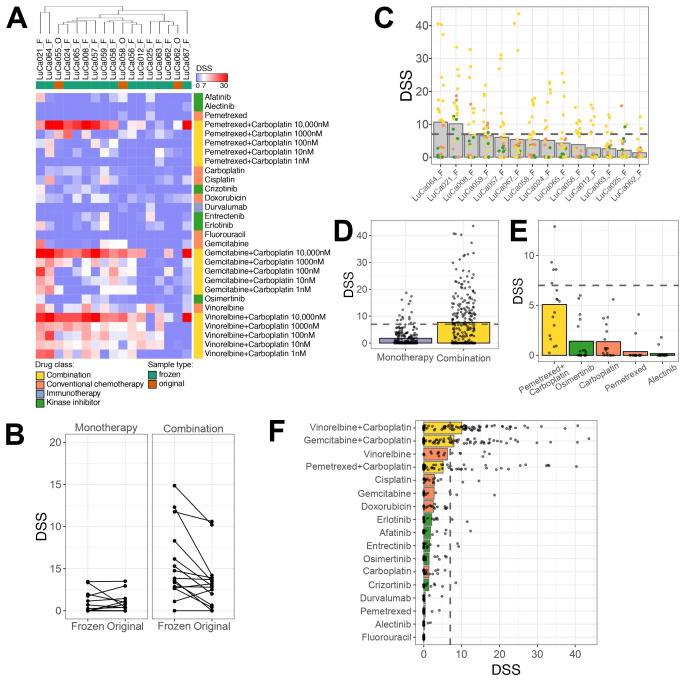
Drug sensitivity testing reveals unique drug response profiles. (**A**) Heatmap representing DSS calculated based on spheroid volume measured after 72 h post-treatment. Unsupervised hierarchical clustering of samples was performed using the Euclidean distance metric. Samples represented on top of the plot indicated with F represent frozen samples, while O indicates original samples. Color codes for drug classes and samples are provided in figure legend. (**B**) Comparison of drug responses between original and frozen samples, presented as DSS after 72 h of treatment. Each line indicates an individual drug. (**C**) Overview of drug response for each tested sample, representing only viable frozen samples. Dots indicate individual drugs or combination condition. (**D**) Comparison of DSS values between responses to monotherapy and combination treatment across tested samples. (**E**) Drug response to clinically relevant drugs; each dot represents a sample. (**F**) Overview of the most effective monotherapy or drug combinations ranked by response. Each dot represents an individual sample; bars colored according to drug classes.

To evaluate individual patient drug sensitivity profiles, we further used uncultured PE samples from lung cancer patients, commonly observed at advanced disease stages [26]. To assess the compatibility of the DET3CT platform for drug testing using PEs, we further performed DST on 27 samples obtained from 21 patients against the LuCa library. The PE sample cell viability was consistently high (>80%) prior to drug testing. Overall, 17 out of 27 samples (65.5%) showed sufficient assay quality, defined as a Z′-factor ≥0.2, and were therefore included in further analysis. A significant proportion of the samples (10 out of 27, 37.04%) failed the assay quality control threshold and were therefore excluded from further analysis (Supplementary Figure S3A, Supplementary Table S4). The samples displayed diverse spheroid morphologies (Supplementary Figure S3B). In addition, the majority of samples with poor assay quality also showed a high proportion of dead cells, which likely impaired their ability to form spheroids and proliferate over time (Supplementary Figure S3C). Samples with sufficient material were tested both fresh and frozen to assess the impact of sample storage on drug response. Among the five tested sample pairs, two demonstrated good assay quality in both original and frozen samples, while the remaining three failed the quality check. The sample pairs that passed assay control exhibited similar spheroid morphology under both fresh and thawed conditions. Overall hierarchical clustering of the drug responses revealed that original (fresh) and frozen samples clustered closely together, indicating similar drug sensitivity profiles (Figure 4A). Furthermore, no statistically significant differences in drug sensitivity were observed between the original and frozen samples for either single-agent (*p* = 0.909) or combination treatments (*p* = 0.145). Nevertheless, the frozen samples exhibited slightly higher sensitivity to drug combinations, with a differential DSS of 2.19 (Figure 4B). The absence of significant differences between the original and frozen conditions indicates that frozen samples have similar drug sensitivity as the original fresh samples. Therefore, the majority of the drug testing was performed on frozen samples thereafter.

Overall, the tested samples exhibited patient-specific drug response profiles, with LuCa064 and LuCa021 showing the highest drug sensitivity (Figure 4C, Supplementary Table S5). While most single-drug treatments did not elicit strong effects across the majority of the samples, combination treatments showed statistically higher sensitivity compared to single drugs based on DSS alone (*p* > 0.0001) (Figure 4D and Figure S2D,E). Among the drugs tested, carboplatin, pemetrexed, alectinib and osimertinib are used in clinical settings for lung cancer treatment (Figure 4E). Alectinib, a targeted therapy for tumors with ALK pathway alterations, was administered to one patient, LuCa057, with no effect. This lack of response was reflected both in the ex vivo assay and the clinical outcome, as this patient had a PFD of 0 months and developed recurrent PE within 2 months. Interestingly, two other patients, LuCa055 and LuCa066, who had no detectable mutations, demonstrated a modest response to alectinib (DSS > 7) ex vivo. These findings suggest that such patients might potentially benefit from ALK-targeted therapy, despite the absence of genetic alterations. Osimertinib, typically used for patients with EGFR mutations, displayed limited activity ex vivo. Among all the tested samples, three patients, LuCa008, LuCa059 and LuCa067, showed the highest response to this drug. However, the DSS values were low (DSS = 6, 4.1, 3.6, respectively). LuCa065, a patient who received a combination of carboplatin/pemetrexed/osimertinib in the clinic, did not show ex vivo sensitivity to any of these drugs as monotherapies, despite carrying an EGFR mutation. While treatment with carboplatin and pemetrexed alone did not show significant responses as single agents ex vivo, we observed a strong drug response to the combination of carboplatin and pemetrexed. Three patients, LuCa056, LuCa065 and LuCa012, received this dual combination therapy in the clinic. For LuCa065, pemetrexed and 10 µM carboplatin emerged as the top hit in the ex vivo testing, with a DSS of 25.5. Although LuCa056 and LuCa012 responded more strongly to gemcitabine or vinorelbine in combination with carboplatin than to pemetrexed with 10 µM carboplatin, the response to the latter was of an adequate and sufficient sensitivity (DSS = 8.7 and 9.8, respectively). Overall, high DSS values for the pemetrexed and carboplatin combination were observed in 5/15 frozen samples, suggesting that this treatment could be of benefit to more patients than currently treated with this combination.

Among the other tested drugs, vinorelbine exhibited the highest efficacy as a single drug, showing efficacy in 7/15 frozen samples, with even greater response under combination treatment with carboplatin (Figure 4F). To better understand the influence of how the combination treatment contributed to the response ex vivo, we determined the synergy between the effects of the drugs, as described in the Materials and Methods section. The results reveal that five patients (LuCa056, LuCa064, LuCa062, LuCa012 and LuCa058) showed strong synergy (ZIP > 10) for all three tested combinations (Supplementary Table S6). LuCa008 demonstrated strong synergy only for the combination of gemcitabine and carboplatin (ZIP = 35.0). Four other patients (LuCa057, LuCa063, LuCa067 and LuCa024) had additive effects (0 < ZIP < 10) to all the combination treatments. Two patient samples (LuCa065 and LuCa021) responded in opposite ways to the combination treatment, with additive effects for one and antagonistic for others. Only one patient sample, LuCa025, showed a consistent antagonistic response to all the tested drug combinations (ZIP ≤ −10). Taken together, this indicates that the DET3CT platform can be applied for the drug-sensitivity testing of cancer cells in PE samples from lung cancer patients. However, the success of the assay may rely on sample quality, cell proliferation and overall viability of the cells in the sample.

### 3.5. Correlations Between Clinical Outcomes and Drug Sensitivity Score

**Figure 5:**
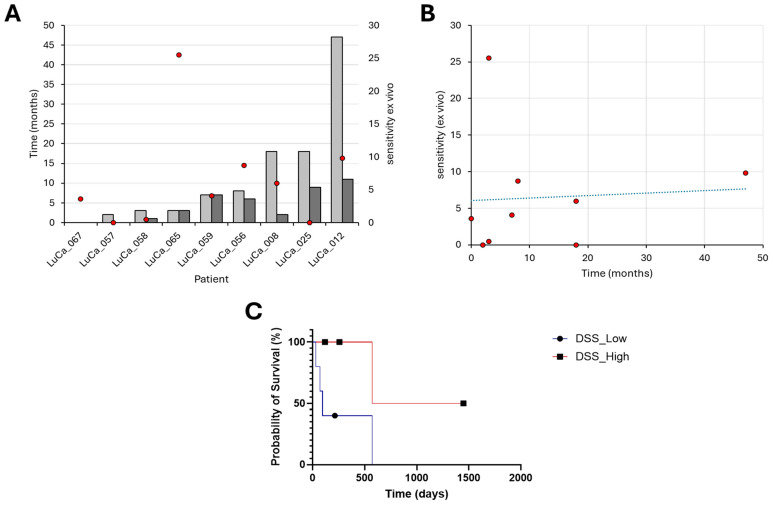
Drug sensitivity of patient-derived cells ex vivo correlates positively with the overall survival of patients in the clinic. (**A**) Overall survival after PE (OS PE) after pleural effusion (light gray) and progression-free survival (dark gray) shown, with corresponding ex vivo DSS scores (red dots), and right panel. (**B**) Correlation of DSS with overall survival after pleural effusion, with drug sensitivity scores (red dots). Spearman correlation coefficient of 0.32, *p* = 0.39. (**C**) Kaplan–Meier curve displays the estimated survival probability between patients and the corresponding overall median DSS (blue line, dot) and above (red line, square).

We excluded twelve patients from further statistical analysis because no treatment was applicable (n = 7), the quality of the cell samples was poor (Z′-factor < 0.2) (n = 3) or no corresponding treatment was included in the design of the LuCa drug library on the DET3CT platform (n = 2). The subgroup of nine patients in which it was possible to establish a correlation between the ex vivo sensitivity score and the clinical effect was composed of patients who had received osimertinib, alectinib or triple combination therapy. For patient LuCa065 who had received a triple treatment of carboplatin/pemetrexed/osimertinib, we included the corresponding DSS value for the combination of carboplatin/pemetrexed. The Spearman correlation analysis showed a moderate positive correlation between the DSS and the OS PE (r = 0.32, *p* = 0.39) (Figure 5A,B). We also evaluated the Spearman correlation coefficient for PFS and the DSS. This showed a moderate correlation with no statistical significance (r = 0.37, *p* = 0.31). To perform a Kaplan–Meier analysis, we first split the group into two subgroups based on the median DSS. The median OS was 94 days in the low DSS group and 1009 days in the high DSS group (Figure 5C). Analysis of these groups with the Mantel–Cox (*p* = 0.127) and Gehan–Breslow–Wilcoxon (*p* = 0.093) tests showed no statistically significant difference, with corresponding chi squares of 2.332 and 2.824. The hazard ratio (Mantel–Haenszel method) for the low vs. high DSS group is 3.998 (95% CI: 0.6751–23.68), while the log-rank test estimates a hazard ratio of 4.630 (95% CI: 0.7984–26.85). These analyses suggest a trend toward worse survival in the low DSS group; however, the small sample size limits the statistical power.

### 3.6. Ranking the Mutated Genes According to Potential Driver Roles in Cancer

**Figure 6:**
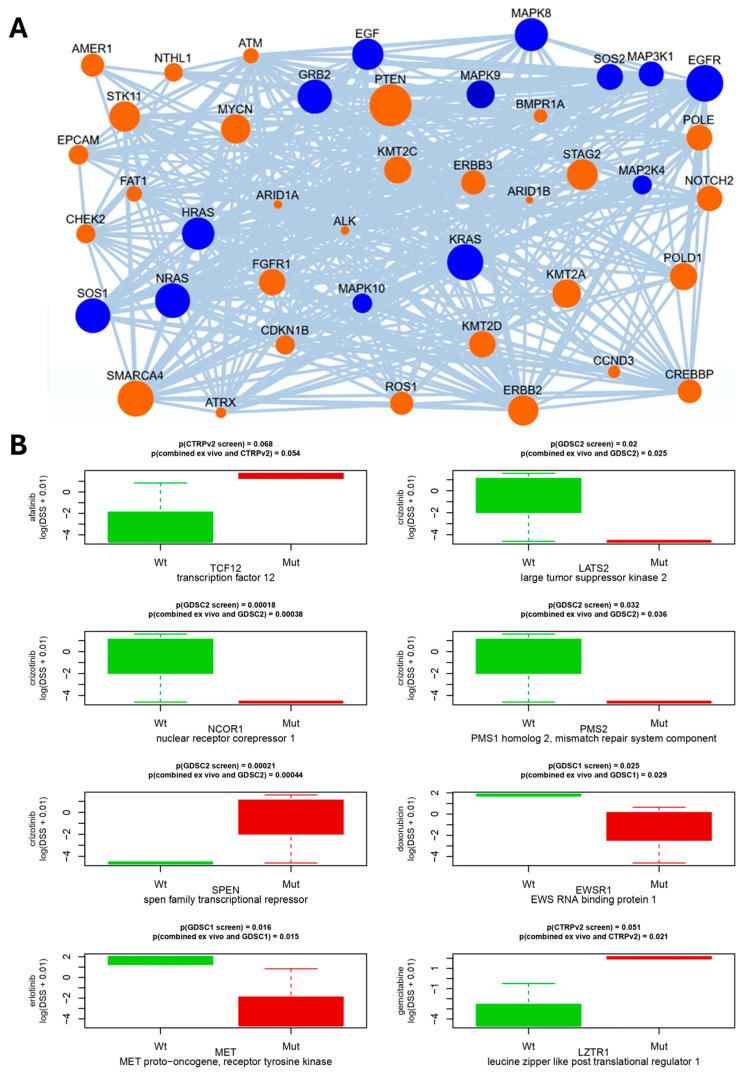
Network connectivity of putative driver genes for patient LuCa058. (**A**) Links between the detected mutations in the same genome and KEGG_medicus “Reference_egf_egfr_ras_jnk_signaling” pathway, one of 38 pathway components in the machine learning model for PathReg score. Edges denote network links between genes and/or proteins reported by STRING and PathwayCommons resources. Node sizes represent node degrees (log of number of links of the gene in the global network, accounted for by NEA algorithm). In the networks, mutated genes (orange) and biochemical signaling pathway genes (blue) are shown as indicated. (**B**) Matching correlations between drug sensitivity/resistance to gene mutations of the three public drug screen datasets (GDSC1, GDCS2, CTRPv2.0) with respect to cell line mutation profile known from CCLE exome sequencing, showing presence of wild type (green) and mutated (red) genes indicated, together with *p*-values, above the graphs. *p*-values refer to comparisons between known cell line mutation profiles to either (top) previously published drug sensitivity/resistance profiles or the combined (lower line) with our ex vivo sensitivity/resistance data, as described in the section on statistics.

In order to determine to which degree genomic mutations could drive the growth and drug resistance of the cancers, we analyzed five LuCa genomic samples from LuCA patients treated with osimertinib. They are represented by sets of mutations derived from the previous panel targeted sequencing, with the method NEAdriver, as described in detail in the Materials and Methods section. Briefly, the NEAdriver algorithm can evaluate a putative driver role of each mutated gene from both global and local sample-specific network contexts. We thereby identified potential driver mutations, which mainly included tyrosine receptor growth factors, growth factor receptors, mitogenic intracellular signaling pathways and cell-adhesion molecules, such as ERBB2, EGFR, FGFR, RAS-ERK signaling, MDM-p21, integrin alpha and beta, talin, vinculin and cohesin (Figure 6A), all molecules are known to regulate cancer. Key tumor suppressor genes, such as PTEN, and well-known oncogenes, such as Notch and Myc, showed up as major central mutations, and all variants of the most commonly mutated oncogene in human cancers, Ras, were predominant pathway genes (Figure 6A, Supplementary Table S7). To further determine if the observed DSS profiles could be linked to specific mutations, we analyzed if these mutations were enriched in previously published in vitro drug screen datasets. Due to a low sample size (n = 5), three large in vitro drug screen datasets have been taken into account [27]. For this purpose, we used a database of pre-computed molecule correlates of anti-cancer drug resistance versus omics profiles obtained from the Cancer Cell Line Encyclopedia [28]. Thereby, we identified eight genes in which mutations correlated to our observed in vitro drug resistance to the same drugs (Figure 6B). These genes included transcription regulators (TCF12, NCOR1, SPEN), kinases (LATS2, MET), RNA-binding protein (EWSR1), substrate-specific adapter of E3 ubiquitin–protein ligase complex (LZTR1) and endonuclease (PMS2).

## 4. Discussion

Primary pleural mesothelioma and metastatic lung cancer are frequently diagnosed at advanced stages, often accompanied by malignant PE, which offers an opportunity for minimally invasive sampling. Despite advances in molecular profiling, only a subset of patients benefits from targeted therapies, which underscores the urgent clinical need for functional tools to guide treatment. Ex vivo drug sensitivity testing has emerged as a promising complement to genomic approaches, as it offers the potential to personalize therapy by a direct evaluation of tumor cell responses to drugs. In this study, we evaluated the correlation between the clinical outcome and the ex vivo cell sensitivity to the drugs currently recommended for NSCLC treatment by implementing an unbiased and double-blind approach. The procedure granted a significant advantage in the context of clinical research, and, in addition, it added methodological rigor by minimizing bias both during the generation and interpretation of data. Hence, the major methodological strength of our functional precision medicine approach is that it minimized confounding factors and allowed a robust evaluation of the correlation between clinical and drug screening outcomes. We could overcome the limitations of molecular profiling that focus only on actionable mutations, without taking into account the complex mutational landscape and signaling pathways that can suppress the efficacy of clinically predicted treatment that usually targets only one driver mutation.

Here, we tested the sensitivity to clinically relevant drugs cells from 21 patients diagnosed with malignant pleural effusion, which we had selected based on clinical and experimental inclusion criteria, such as molecular profile and availability of material for further drug screening analysis. The cohort reflected the typical demographic and clinical characteristics of advanced lung cancer. It is noteworthy that despite the identification of molecular targets in the patients, only 43% received targeted therapy, while 24% were treated with conventional chemotherapy and immune checkpoint inhibitors, and 33% remained untreated. While the DET3CT drug testing platform was originally developed and validated with fresh patient sample material, we successfully adapted this assay for frozen samples. This modification enables drug sensitivity testing to be performed at a later time-point when a fresh sample is not available. This can provide major advantages, as, for example, the analysis can be conducted after omics characterization of the sample, which can inform patient-specific drug combinations. Furthermore, frozen samples enable centralized ex vivo testing across multiple clinical sites, which potentially can simplify clinical implementation. To evaluate the adapted platform, we tested the NSCLC-derived PDC model LuCa011 along with two well-characterized lung cancer cell lines, HCC827 and H1869, to control the potential effects related to culture conditions. The media used for the ex vivo assays contain EGF, which previously has been reported to suppress the response to EGFR inhibitors by activating the MAPK pathway [29,30]. Nonetheless, the presence of EGF did not alter the sensitivity of the tested cells to EGFR inhibitors, which supports the robustness of our assay. In this context, it is, however, important to keep in mind that none of the samples of the patients who received osimertinib in the clinics showed sensitivity in the drug screening, which may be due to the presence of EGF in the medium. Further, we applied the assay on the sample LuCa011, which lacked the most common NSCLC-associated mutations, yet carried several rare alterations, such as a loss-of-function mutation in SMARCA4. Interestingly, these cells showed notable sensitivity to vinorelbine, which align with the previous observation that reduced SMARCA4/BRG1 expression may predict a favorable response to cisplatin/vinorelbine [31]. These cells did not show a strong response to cisplatin or to the other conventional chemotherapies tested but were instead sensitive to targeted therapies, e.g., afatinib and crizotinib, a sensitivity which could not be predicted based on the mutational profile of this sample. This highlights the value of functional drug testing platforms, such as DET3CT, which can identify effective therapeutic options even when genomic data lacks actionable mutations.

To determine the correlation between the cell sensitivity ex vivo and the clinical outcome, we included only the patients who received a corresponding treatment in the clinical setting and for whom the screening platform provided evaluable drug response data. Samples that did not meet the predefined quality control on the platform were also excluded from the subsequent statistical analysis. As a result, out of the initial cohort of 21 patients, 9 were included in the final correlation analysis. For these nine patients, we observed a moderate positive correlation between OS PE and the drug sensitivity score ex vivo. The comparison between PFS and the ex vivo response showed a similar trend. To understand the variability of data, it is important to consider the heterogeneity of patient samples and data, such as the large disparity of total follow-up time between patients. As most of our patients had a very long follow-up, several patients only attended a limited follow-up due to late enrollment into this study. At the last follow-up, four out of nine patients (LuCa012, LuCa056, LuCa059, LuCa065) were still alive and thus censored in the survival analysis. In particular, LuCa065 was enrolled only three months before the cutoff date, which resulted in a short OS PE value, despite presenting a relatively high sensitivity to combination therapy. This results in a moderate correlation, which did not achieve statistical significance. For the Kaplan–Meier survival analysis, we investigated the difference between the two subgroups based on the median DSS. We observed a trend associating the higher DSS with a better probability of survival for the patient. While such analysis can be performed on small datasets, the statistical robustness is considerably limited. It is essential to note that, as a consequence of these parameters, as well as heterogeneity and the size of the cohort, the results are mainly descriptive and may not reflect real-world outcomes. This low inclusion rate introduces a high risk of selection bias and limits the statistical power of this study, increasing the likelihood of type II errors and reducing the ability to draw more generic conclusions. Specifically, the patients received different therapies, and most of the selection for correlation was carried out post hoc due to technical reasons related to the ex vivo screening procedures. The resulting survival curves must therefore be interpreted with caution, as the statistical power of log-rank testing is severely reduced in such a limited cohort. Nevertheless, we observed a tendency toward a positive association between longer survival and sensitivity to drugs ex vivo.

To evaluate the cofactors that may impact the correlation between DSS and the clinical outcome, exploratory Fisher’s exact tests were conducted, including parameters such as smoking status, age or sex of the patient. However, these analyses did not reveal any significant associations with the clinical parameters. This is also probably due to the heterogeneity and the size of the cohort. Additionally, we analyzed five lung cancer samples using mutation data obtained from prior panel-based targeted sequencing to gain insight into the extent to which genomic alterations might contribute to tumor progression and resistance to osimertinib. The NEAdriver approach used in this study assesses the potential driver role of each mutated gene by integrating both global and sample-specific gene network contexts, allowing us to estimate the functional relevance of individual mutations in each patient’s tumor. We identified potential driver mutations with unexpected factors, such as integrin alpha and beta, talin, vinculin and cohesin. These results show the complexity of cells and highlight the oversimplification of standard molecular analysis currently performed in the clinics. While only targeting the identified mutations (in this case EGFR), we omit other potential drivers that may provide resistance to the proposed therapy. This aligns with our proposed protocol that overcomes this oversimplification. These results may be interesting to explore in future studies to evaluate resistance mechanisms or to characterize unexplored driver cancer mutations.

Among the patients who received treatment, three did not show clinical improvement, which is consistent with the fact that the samples from these patients showed resistance to drugs in the ex vivo drug test. Two of them (LuCa058 and LuCa067) were treated with osimertinib and showed a DSS corresponding to 0.5 and 3.6, respectively. Patient LuCa057 received alectinib and had a DSS of 0. Interestingly, all three samples demonstrated a sensitivity for different drug combinations in the screening assay, with the highest sensitivity for the chemotherapy combination of vinorelbine/carboplatin. This highlights the fact that despite the patients harboring sensitizing EGFR- or ALK mutations, they were not responsive to the targeted treatment typically proposed based on their mutation profile. It is possible that this is due to other potential gatekeeper mutations or resistance mechanisms that have not been taken into account. Taken together, this suggests that a drug sensitivity screening should be used complementary to molecular profiling for effective personalized treatment. Another possibility would be to apply a network-based method accounting for mutation composition of individual cancer genomes. Since this approach should consider more rare driver events, it will require sequencing of the full exome rather than a limited panel of known cancer driver genes.

The patient LuCa065 experienced clinical benefit after receiving triple therapy of carboplatin/pemetrexed/osimertinib. As the DSS for carboplatin/pemetrexed was 25.5, and 0 for Osimertinib, this suggests that although the tumor had an EGFR mutation, the combined treatment with carboplatin and pemetrexed may have been sufficient, and the TKI-targeted treatment with osimertinib did not account for the clinical benefit. The patient is reported to harbor a mutation in EGFR, i.e., an EGFR exon 19 deletion, which is commonly sensitive to the first or second generation of TKI. It is possible that the long-term treatment of a third-generation TKI, such as Osimertinib, has exposed the tumor in this patient to a selective pressure that promoted the development of epithelial to mesenchymal transition [32], as well as resistance mutations [33]. Another patient, LuCa025, who harbors the sensitizing EGFR L858R mutation, experienced clinical benefit from osimertinib; however, this was not reflected in the DSS score. In fact, with the exception of vinorelbine, we observed generally poor sensitivity to the different drugs tested. Moreover, the analysis of the synergy of drug combinations showed, in general, antagonistic effects. Taken together, this underlines the possibility that the presence of other mutations that have not been identified may also play an important role. It is also important to take into account that pharmacodynamic events, non-tumor cells and the tumor microenvironment are also likely to modulate the response to drugs [34,35]. Our study highlights, therefore, that caution should be taken to not over-simplify the cellular processes that support tumor growth and spread. Specifically, we note the importance of understanding and considering the complexity of the biological selection processes that occur during tumor development and result in targetable phenotypes. This reiterates that it is important to keep in mind that selection pressure is exerted on cell phenotypes, and not on the genotype. Much remains to be understood about the regulation of how mutations in the genome are or are not translated into functional phenotypes in cancer cells. Genomic mutations are often not sufficient to drive tumors, and a permissive environment is also required for tumor development and growth [34], and this environment also modulates the response to the chemotherapeutic agents [35]. To address this complexity, multi-omic analyses can be combined with functional drug sensitivity testing in model systems that include a tumor-mimicking stroma to support clinical decision-making [36]. Recent technological advances now enable multi-omic analyses to be performed on very small specimens, which can broaden their clinical applicability. Given the importance of the tumor environment, a major advantage of the use of pleural effusion is that the cancer cells therein grow in a liquid tumor environment in which the physical and spatial cues are similar to the suspended 3D cell culture used in our sensitivity testing, which offers a testing model that mimics the in vivo situation better than testing on cancer cells from solid tissue. Another important advantage of pleural effusions is that, as demonstrated in this study, it offers a valuable opportunity to reduce the need for invasive procedures in patients with advanced-stage disease. The major challenge encountered during this study is the high number of patients excluded, both on the clinical and experimental side. In the clinical setting, several patients had poor performance status and were not eligible to receive therapy or did not have enough biological material to be included in laboratory testing. On the laboratory side, various samples could not be tested due to technical or logistic reasons, which affected the quality of the samples. Some samples had a low tumor representation or were excluded due to the poor quality of the cells in 3D culture. To overcome these obstacles in future studies, we plan to explore procedures for rapid sample procurement and clear data exchange with oncologists at early decision points. Analogous to hematological procedures, a dedicated team could ensure the sample handling and processing, playing an important role in minimizing delays in subsequent analysis. Notably, this study was initiated from a diagnostic perspective. While our approach enabled an adaptation of a drug screening platform based on current national recommendations for the treatment of NSCLC Stage IV, it may not optimally align with the clinical realities of oncological care. A prospective study initiated and designed from clinical oncology, based on clinical eligibility, could potentially overcome several limitations that we encountered.

One important limitation of our approach is the lack of immune cell components in the assay. This limits the assessment of immune-mediated mechanisms of action, such as antibody-dependent cellular cytotoxicity (ADCC) or T-cell-mediated responses. It is particularly relevant when evaluating therapies relying on the immune response, including monoclonal antibodies and immune checkpoint inhibitors [37]. To address this, several groups have developed co-culture systems incorporating autologous peripheral blood mononuclear cells (PBMCs) or purified immune cell subsets alongside patient-derived tumor spheroids or organoids [38,39,40]. While technically more complex, such strategies provide a more comprehensive understanding of drug efficacy in an immune-competent context. Recently, it has been shown that CD45+ cells are present in ovarian cancer ex vivo samples, which supports that our assay maintains different components of the tumor microenvironment [15]. In our study we did not evaluate the immune cell population present in the extracted pleural effusion from the patients. Future prospective studies should include optimization of the protocol to provide a predictive value for immunomodulatory agents.

Taken together, our findings suggest that ex vivo drug screening adds value by predicting the clinical outcome, especially if combined with targeted sequencing and network analysis and a comprehensive characterization of the disease, and also highlights limitations, such as limited inclusion of molecular data and a small and heterogeneous patient cohort with unidentified variabilities.

## 5. Conclusions

Our findings progress the current conceptual framework and technical approach used for ex vivo sensitivity analysis and highlight limitations and recommendations for future prospective studies. The ex vivo drug sensitivity screening of pleural-effusion-derived cells presents a promising opportunity to identify treatment options and enhance the future management of cancers that metastasize to pleural effusion, which expands the potential for personalized oncology. Thereby, our study contributes to the urgently needed improved, new personalized treatments to radically improve the clinical outcomes for lung cancer and mesothelioma patients.

## Figures and Tables

**Table 1:** Baseline patient characteristics.

Variables	Cohort (N = 21)
**Sex**	
Male	9 (42.9%)
Female	12 (57.1%)
**Smoking status**	
Non-smoker	7 (33.3%)
Former smoker	11 (52.4%)
Current smoker	3 (14.3%)
**Histology**	
Adenocarcinoma	19 (90.5%)
Mesothelioma	2 (9.5%)
**PD-L1 expression**	
Low (<1%)	9 (42.9%)
Intermediate (1–49%)	6 (28.6%)
High (≥50%)	2 (9.5%)
Not tested	4 (19.0%)
**Genetic mutation status**	
No actionable mutations	5 (23.8%)
Actionable mutations	12 (57.1%)
*EGFR*	7 (33.3%)
*BRAF^V600E^* *BRAF^V600G^*	1 (4.8%) 1 (4.8%)
*RET*	2 (9.5%)
*ALK*	1 (4.8%)
Not tested	3 (14.3%)
Non-actionable mutations	1 (4.8%)
**Age at malignant pleural effusion diagnosis (years)**	
Median (IQR; range)	76 (36–91)
**Debut with malignant pleural effusion**	11 (52.4%)
**TNM stage at cancer diagnosis**	
Stage 1	6 (28.6%)
Stage 2	0 (0.0%)
Stage 3	2 (9.5%)
Stage 4	13 (61.9%)
**Death**	14 (66.7%)
**Median follow-up time**	10.0 months (0–196)

The characteristics and variables of the patients used in the study, with acronyms as follows: programmed death-ligand 1 (PD-L1), epidermal growth factor receptor (EGFR), B-Raf proto-oncogene (BRAF), rearranged during transfection (RET), anaplastic lymphoma kinase (ALK), interquartile range (IQR).

**Table 2:** Patient treatments and outcomes.

Variables	Cohort (N = 21)
**Type of therapy received after malignant pleural effusion diagnosis**	
Targeted therapy	9 (42.9%)
Osimertinib	5 (23.8%)
Alectinib	1 (4.8%)
Selpercatinib	1 (4.8%)
Pralsetinib	1 (4.8%)
Trametinib/Dabrafenib	1 (4.8%)
Chemotherapy combinations	5 (23.8%)
Carboplatin/Pemetrexed/Bevacizumab	2 (9.5%)
Carboplatin/Pemetrexed/Pembrolizumab	1 (4.8%)
Carboplatin/Pemetrexed/Cemiplimab	1 (4.8%)
Carboplatin/Pemetrexed/Osimertinib	1 (4.8%)
No treatment	7 (33.3%)
**ECOG PS at therapy initiation**	
0	3 (14.3%)
1	9 (42.9%)
2	1 (4.8%)
3	3 (14.3%)
Clinical benefit *	(N = 14)
Yes	10 (71.4%)
No	3 (21.4%)
Not evaluable	1 (7.1%)
Median PFS *	
Cohort	6 months
Patients with targetable mutations	5 months
Patients without targetable mutations	6 months
**Median OS**	
Cohort	10 months
Patients with targetable mutations	14 months
Patients without targetable mutations	8 months
**Median OS from malignant pleural effusion diagnosis**	
Cohort	3 months
Patients with targetable mutations	3.5 months
Patients without targetable mutations	3 months

The fraction of patients who received drug treatments and clinical outcomes, with abbreviations as follows: Eastern cooperative oncology group performance status (ECOG PS), progression-free survival (PFS), overall survival (OS). * indicates patients who received treatment.

**Table 3:** Detailed patient characteristics, histological subtype, molecular status, personalized treatment and clinical response applicable.

Patient ID	Age PE	Sex	Diagnosis	Stage	Mutation	Locus	Therapy	PFS	OS	OS PE
LuCa008	91	F	AC	1A2	1	EGFR (Exon 19, Glu746_Ala750del)	Osimertinib	2	40	18
LuCa009	52	F	AC	4A	0	-	Carboplatin/Pemetrexed/Pembrolizumab	6	36	34
LuCa011	73	M	AC	4B	N/A	N/A	N/A	N/A	0	0
LuCa012	73	M	PM	4	N/A	N/A	Carboplatin/Pemetrexed/Bevacizumab	11	48	47
LuCa020	76	F	AC	4A	1	BRAF (V600E)	Dabrafenib/Trametinib	21	23	23
Luca021	69	F	AC	4B	1	RET fusion	Pralsetinib	10	20	20
LuCa024	84	F	AC	4A	0	-	N/A	N/A	0	0
LuCa025	79	F	AC	4A	1	EGFR (Exon21, L858A)	Osimertinib	9	18	18
LuCa054	84	F	AC	4A	1	EGFR (Exon21, L858A)	N/A	N/A	0	2
LuCa055	75	F	AC	1B	0	-	N/A	N/A	8	2
LuCa056	71	M	AC	1A3	0	-	Carboplatin/Pemetrexed/Cemiplimab	6	22	8
LuCa057	80	M	AC	1A2	1	ALK fusion	Alectinib	0	36	2
LuCa058	80	F	AC	3A	2	EGFR (Exon 18 and Exon 21)	Osimertinib	1	46	3
LuCa059	89	F	AC	1A2	2	EGFR (Exon 20,T790M), EGFR (Exon 21, L858A)	Osimertinib	3	192	7
LuCa061	75	M	AC	4B	0	-	N/A	N/A	2	2
LuCa062	88	M	AC	4A	1	BRAF (Exon 15, V600G) IDH1 (Exon 4, A132C)	N/A	N/A	5	5
LuCa063	39	F	AC	4B	1	Fusion KIF5B(16)-RET(12)	Selpercatinib	10	10	4
LuCa064	70	M	AC	3A	1	FGFR3	N/A	N/A	7	1
LuCa065	36	M	AC	4A	1	EGFR (Exon 19, G746_A750del)	Carboplatin/Pemetrexed/Osimertinib	3	3	3
LuCa066	79	M	PM	1A	N/A	N/A	Carboplatin/Pemetrexed/Bevacizumab	0	4	3
LuCa067	81	F	AC	4B	1	EGFR (Exon 19 del)	Osimertinib	0	0	0

Patient ID; age at pleural effusion-derived diagnosis (Age PE); sex: male (M), female (F); adenocarcinoma (AC); pleural mesothelioma (PM); diagnosis, stage, identified mutations, received therapy, progression-free survival (PFS) overall survival (OS) and overall survival after pleural effusion recovery (OS PE) are indicated in months; N/A: not available.

## Data Availability

The original contributions presented in this study are included in the article/Supplementary Materials. Further inquiries can be directed to the corresponding author.

## Supplementary Materials

The following supporting information can be downloaded at: https://www.mdpi.com/article/10.3390/cancers17142363/s1, Figure S1: Training model for Network connectivity of putative driver genes. (A) Links between gene ARID1A and the set of putative mutations in genome LuCa058, used to calculate MutSet score for ARID1A. (B) Training and testing PathReg model for lung adenocarcinoma on respective LUAD TCGA cohort, including the pathways in the model and weight coefficients. One third of the genes (shown in orange) were withheld while the training step and used only for testing. Rank correlations represent goodness of the model fit; Figure S2: Copy number variation (CNV) profile of the sections of the genome that are repeated within the LuCa011-PDC cells; Figure S3: (A) Overview of sample assay quality expressed as Z’-factor values; red dots indicate samples that were excluded from further analysis. (B) Cells that displayed divers spheroid morphologies or (C) Cells that displayed a high proportion of death. (D-E) Ranking of drug responses under (D), single drug and (E), drug combination treatment. Data represented as DSS values after 72 h of drug treatment, with each dot representing an individual drug or combination; Table S1: LuCa library containing drugs, used concentrations and annotations, Table S2: List of samples and patients used for DET3CT platform testing; Table S3: High impact mutations reveald from target sequencing, when validating LuCa011 model. Figure representing CNVs in Figure S1; Table S4: Drug sensitivity testing assay quality represented with Z′; Table S5: Drug sensitivity scores for the sampled that passed QC. Values represent DSS calculated based on spheroid volume measurement; Table S6: Drug synergy results for tested samples, represented as ZIP synergy scores; Table S7: NEAdriver method evaluating and ranking the mutated genes according to potential driver roles.
