# Supplementary material for: Ex Vivo Drug Sensitivity of Pleural Effusion-Derived Cells from Lung Cancer and Pleural Mesothelioma Patients Is Linked to Clinical Response
Source: Cancers (Basel). 2025 Jul 16;17(14):2363. doi: 10.3390/cancers17142363 (PMC12293284; doi:10.3390/cancers17142363)
Supplement: Supplementary file 1 [file cancers-17-02363-s001.zip › cancers-3706129 Supplementary Figures edited.pdf]

Supplementary Materials: Ex vivo drug sensitivity of pleural-effusion-derived cells from lung cancer and pleural mesothelioma patients predicts clinical response.

Rita Hutyra-Gram Ötvös, Hanna Krynska, Greta Gudoityte, Marcus Skribek, Anca Oniscu, Olena Berkovska, Katharina Strauss, Jenny Zipprick, David Tamborero, Andrey Alexeyenko, Annica Karin Britt Gad, Brinton Seashore-Ludlow and Katalin Dobra

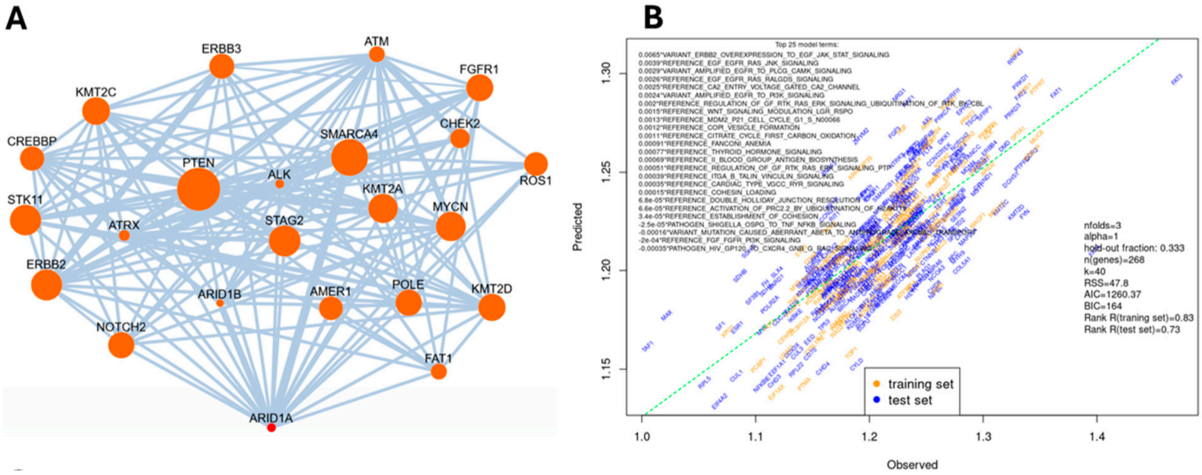

Supplementary Figure S1. Training model for network connectivity of putative driver genes. A, Links between gene ARID1A and the set of putative mutations in genome LuCa058, used to calculate MutSet score for ARID1A. **B**, Training and testing PathReg model for lung adenocarcinoma on respective LUAD TCGA cohort, including the pathways in the model and weight coefficients. One-third of the genes (shown in orange) were withheld from the training step and were used only for testing. Rank correlations represent goodness of the model fit.

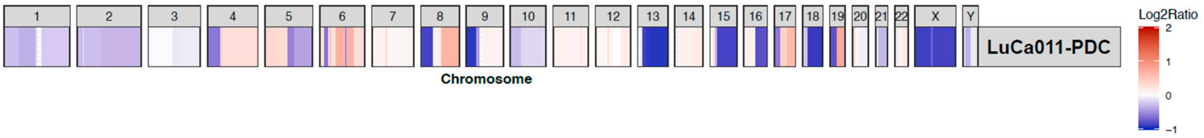

Supplementary Figure S2. Copy number variation (CNV) profile of the sections of the genome that are repeated within the LuCa011-PDC cells.

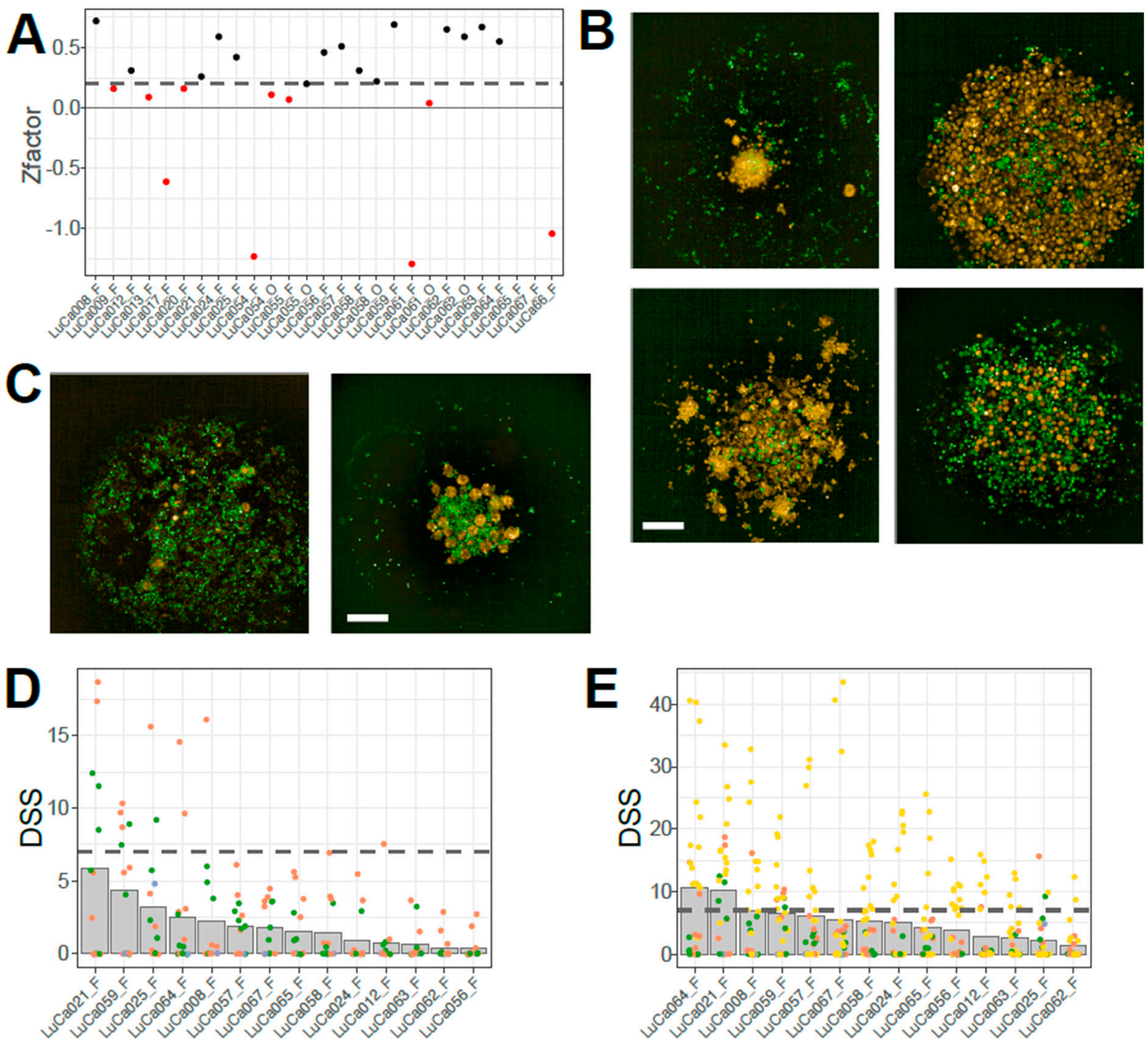

Supplementary Figure S3. A, Overview of sample assay quality expressed as *Z*′-factor values; red dots indicate samples that were excluded from further analysis. **B** Cells that displayed divers spheroid morphologies or, **C**, cells that displayed a high proportion of death. **(D-E)** Ranking of drug responses under, **D,** single drug and, **E,** drug combination treatment. Data represented as DSS values after 72 h of drug treatment, with each dot representing an individual drug or combination.
